# Quantitative screening of geranylgeranoic acid in selected plant-based foods using LC/MS/MS

**DOI:** 10.3389/fnut.2025.1652270

**Published:** 2025-08-12

**Authors:** Yuki Tabata

**Affiliations:** Department of Nutritional Health, Nutritional Biochemistry, Faculty of Wellness Studies, Kwassui Women's University, Nagasaki, Japan

**Keywords:** geranylgeranoic acid, dietary isoprenoids, lipid mediators, cancer chemoprevention, hepatic carcinogenesis, LC/MS/MS, plant-derived compounds, nutritional biochemistry

## Abstract

Geranylgeranoic acid (GGA), a bioactive acyclic isoprenoid, has been developed as a preventive agent against recurrent hepatocellular carcinoma. While previous studies have identified GGA in certain medicinal herbs, such as turmeric, its presence in other commonly consumed plant-based foods remains largely unexplored. In this study, we screened 14 plant-based food items using a validated LC/MS/MS method to quantify their GGA content. Among the tested samples, turmeric powder exhibited the highest GGA concentration (20.2 ± 8.25 ng/g dry weight), consistent with previous findings. GGA was also detected in several nuts, including almonds (Italy: 7.59 ± 2.45 ng/g; USA: 6.48 ± 1.28 ng/g), cashew nuts (4.12 ± 1.12 ng/g), and pistachios (3.48 ± 0.95 ng/g). Importantly, azuki beans (7.21 ± 2.12 ng/g) and soybeans (1.21 ± 0.29 ng/g) were also found to contain GGA, suggesting that some legumes may serve as additional dietary sources of this compound. In contrast, GGA was not detected above the limit of quantification for the following seven items: chickpea, walnut, sesame seeds, and dried parsley. These negative results are also informative for defining the boundaries of GGA distribution in plant-based foods. This study provides new data on the occurrence of GGA in plant-derived foods and contributes to a comprehensive understanding of its potential dietary sources.

## 1 Introduction

Geranylgeranoic acid (GGA) was originally developed as an acyclic diterpenoid retinoid (1, 2). It exhibits ligand activity for retinoic acid receptor (RAR) and retinoid X receptor (RXR) and can induce differentiation in hepatoma cell lines (3). Subsequently, Shidoji et al. reported that, unlike naturally occurring retinoids, GGA induces non-apoptotic cell death in human hepatoma-derived cell lines at micromolar concentrations. Recently, it has been shown to induce cell death in human hepatoma cells via TLR4-mediated pyroptosis, a form of genetically regulated necrosis associated with inflammatory responses (4, 5).

GGA was later identified as a naturally occurring compound present in medicinal herbs such as turmeric (*Curcuma longa*), which is widely used in traditional Ayurvedic medicine (6). In addition to turmeric, GGA has also been detected in various medicinal herbs such as Schisandra (*Schisandra chinensis*), licorice (*Glycyrrhiza uralensis*), Indian gooseberry (*Emblica officinalis*), and rhubarb root (*Rheum palmatum*), albeit at lower concentrations (6). These findings suggest that GGA may be more broadly distributed across plant species than previously recognized. In a human study, oral intake of turmeric tablets significantly elevated plasma GGA concentrations within 2–4 h, followed by a gradual decrease over the next 8 h, thereby confirming the gastrointestinal absorption of dietary GGA (7). In addition to plant-derived sources, GGA has also been shown to be endogenously biosynthesized in mammals, including humans (8–10). Recently, a study demonstrated a relationship between endogenous hepatic GGA levels and spontaneous hepatocarcinogenesis in male C3H/HeN mice. This mouse strain naturally develops liver tumors at approximately two years of age. We observed a significant age-related decline in hepatic GGA content, with GGA being completely depleted in tumor-bearing livers. Notably, oral administration of GGA at 11 months of age—just prior to the decline in hepatic GGA levels—markedly suppressed the incidence of liver cancer at 23 months, suggesting the potential of dietary GGA supplementation as a chemo-preventive strategy against hepatocellular carcinoma (11).

These results support the potential of GGA as a bioactive nutrient for cancer chemoprevention. Specifically, increasing hepatic GGA levels through dietary intake may offer a promising approach to suppress liver tumorigenesis, forming the basis of a future paradigm of “diet-based liver cancer prevention”.

To date, GGA has only been reported in a limited range of plant materials, notably turmeric. However, turmeric is not widely consumed in large amounts in most populations, especially outside South Asian cuisines, raising questions about its practicality as a dietary source of GGA for the public. Therefore, to evaluate the feasibility of dietary strategies for GGA supplementation, it is essential to expand knowledge on the distribution of GGA in the commonly consumed foods.

This study conducted a targeted screening of 14 commercially available plant-based foods, including nuts, legumes, oilseeds, and herbs, to identify new dietary sources of GGA. The resulting data provide a foundation for the development of nutritional approaches aimed at liver cancer prevention and metabolic health promotion.

## 2 Materials and methods

### 2.1 Chemicals

GGA was prepared by Kuraray Co. (Okayama, Japan) and Kowa Pharmaceutical Co., Inc. (Tokyo, Japan). Acetonitrile (LC/MS grade) and ethanol were purchased from Sigma-Aldrich (St. Louis, MO, United States). Methanol was purchased from Wako Pure Chemical Industries (Osaka, Japan). Chloroform was purchased from Kanto Chemical Co., Inc. (Tokyo, Japan). All other chemicals were of reagent grade.

#### 2.1.1 Sample preparation

Fourteen types of commercially available plant foods (Table 1) were used in powder form.

**Table 1:** List of plant-based food items analyzed for geranylgeranoic acid (GGA) content, along with country of origin and classification.

No.	Common name	Scientific name	Category	Sample form	Country of origin
1	Turmeric Powder	*Curcuma longa*	Spice	Powder	India
2	Almond	*Prunus dulcis*	Nut	Powder	Italy
3	Almond	*Prunus dulcis*	Nut	Powder	United States
4	Cashew Nut	*Anacardium occidentale*	Nut	Powder	India
5	Pistachio	*Pistacia vera*	Nut	Powder	Italy
6	Azuki Bean	*Vigna angularis*	Legume	Powder	Japan
7	Soybean	*Glycine max*	Legume	Powder (kinako)	Japan
8	Hazelnut	*Corylus avellana*	Nut	Powder	Italy
9	Walnut	*Juglans regia*	Nut	Powder	United States
10	Chickpea	*Cicer arietinum*	Legume	Powder	India
11	White Sesame	*Sesamum indicum*	Oilseed	Roasted and ground (surigoma)	Paraguay
12	Black Sesame	*Sesamum indicum*	Oilseed	Roasted and ground (surigoma)	China
13	Dried Mango Powder	*Mangifera indica*	Dried fruit powder	Powder	—(not specified)
14	Dried Parsley	*Petroselinum crispum*	Dried herb	Dried and crushed	—(not specified)

### 2.2 Lipid extraction

Approximately 1 g of each sample was mixed with 10 mL of methanol and allowed to stand overnight at room temperature. The next day, 20 mL of chloroform was added, and the mixture was vortexed to extract total lipids. The mixture was then centrifuged at 3000 × g for 10 min, and the supernatant was collected. This extraction process was repeated three times. The pooled extracts were evaporated to dryness under a nitrogen atmosphere and subsequently redissolved in 1 mL of ethanol.

The ethanolic solution was applied to ethanol-equilibrated C18 solid-phase extraction cartridges (Bond Elute C18; Agilent, Tokyo, Japan), and the flow-through fractions were collected as lipid extracts. These extracts were filtered using a Cosmonice Filter S cartridge (PTFE, 0.45 μm, 13 mm; Nacalai Tesque, Kyoto, Japan) immediately prior to analysis.

#### 2.2.1 Quantitative measurement of GGA contents

Quantification of GGA was performed using a Waters Acquity UPLC system coupled with a tandem quadrupole mass spectrometer (Waters, Milford, MA) operating in multiple reaction monitoring (MRM) mode, as previously described (10). Chromatographic separation was achieved using an Acquity UPLC-HSS T3 column (2.1 mm × 100 mm, 1.8 μm). The mobile phase consisted of acetonitrile (solvent A) and water (solvent B). The gradient elution profile began with an isocratic phase at 74% A, followed by a linear gradient to 100% A, a hold phase, and a return to initial conditions, with a total run time of 19 min at a flow rate of 0.3 mL/min. Nitrogen was used as both cone and desolvation gas, while argon served as the collision gas. Instrumental parameters such as capillary voltage, source and desolvation temperatures, and collision energy were optimized based on a previously validated method for GGA quantification (8).

#### 2.2.2 Quantification of GGA

Quantification of GGA was performed using an external calibration method without the use of an internal standard. Standard solutions of authentic GGA were prepared by serial dilution in the concentration range of 0.15–2.5 pg./injection. The calibration curve was constructed by plotting the peak area against the corresponding concentrations and showed excellent linearity with a correlation coefficient (R^2^) of 0.9969. Quantification of GGA in food samples was based on peak area comparison with the standard curve. Retention time stability and peak shape were confirmed across multiple injections. The calibration curve used for quantification is provided in Supporting Information (Supplementary Figure S1). The limit of detection (LOD) and limit of quantification (LOQ) were determined based on signal-to-noise ratios of 3 and 10 and estimated to be 0.10 and 0.15 pg./injection, respectively. Recovery of the extraction method was not assessed in this study.

## 3 Results

GGA was detected in 7 of the 14 plant-based food items analyzed (Table 2). The highest concentration was observed in turmeric powder (20.2 ± 8.25 ng/g DW), consistent with previous reports. Among the tested samples, GGA was also found in several nuts, including almond (Italy: 7.59 ± 2.45 ng/g; USA: 6.48 ± 1.28 ng/g), cashew nut (4.12 ± 1.12 ng/g), and pistachio (3.48 ± 0.95 ng/g). Additionally, legumes such as azuki bean (7.21 ± 2.12 ng/g) and soybean (1.21 ± 0.29 ng/g) contained measurable levels of GGA, indicating their potential as dietary sources of this compound. These results confirm that GGA is present not only in turmeric but also in nuts and legumes, which are widely consumed in various diets. Representative MRM chromatograms of GGA were obtained using the transition [M-H]^−^ m/z 303 → 98 in negative ion mode. As shown in Supporting Information (Supplementary Figure S2), the retention time and peak shape of GGA detected in cashew nut, azuki bean and almond samples were consistent with those of the authentic standard. The major fragment ion (m/z 98) was also confirmed based on the MS/MS transition, supporting the identification of GGA in the food matrix.

**Table 2:** Concentration of geranylgeranoic acid (GGA) in 14 plant-based food items (ng per g dry weight, mean ± SD).

No.	Common name	GGA content (ng/g DW)	SD
1	Turmeric Powder	20.2	8.25
2	Almond (Italy)	7.59	2.45
3	Almond (USA)	6.48	1.28
4	Cashew Nut	4.12	1.12
5	Pistachio	3.48	0.95
6	Azuki Bean	7.21	2.12
7	Soybean	1.21	0.29
8	Hazelnut	ND	–
9	Walnut	ND	–
10	Chickpea	ND	–
11	White Sesame	ND	–
12	Black Sesame	ND	–
13	Dried Mango Powder	ND	–
14	Dried Parsley	ND	–

All samples were analyzed in triplicate (*n* = 3). “ND” indicates that GGA was not detected above the limit of quantification. GGA: geranylgeranoic acid.

No detectable GGA levels were found in hazelnut, walnut, chickpea, sesame (white and black), dried mango powder, or dried parsley.

## 4 Discussion

In this study, we quantified the levels of GGA in 14 plant-based food items commonly consumed by humans. Consistent with previous reports, turmeric (*Curcuma longa*) contains the highest levels of GGA among the tested samples (6). Notably, we also detected GGA in various nuts, including almonds, cashew nuts, and pistachios. These findings suggest that tree nuts, in addition to being rich in lipids, may serve as practical dietary sources of GGA.

Epidemiological evidence supports the relevance of these results. Two large prospective US cohort studies, the Nurses’ Health Study and the Health Professionals Follow-up Study, have reported a suggestive association between increased intake of tree nuts (averaging 1.25 servings per week) and a reduced risk of hepatocellular carcinoma (12, 13). This protective effect has primarily been attributed to antioxidant constituents such as polyphenols and vitamin E. However, our findings raise the possibility that GGA, a bioactive lipid uniquely found in certain nuts, may also play a contributory role. This hypothesis warrants further exploration in both mechanistic and population-based studies in the future.

Beyond tree nuts, we detected GGA in two types of legumes, azuki beans and soybeans, which are widely incorporated into East Asian diets (14). This is particularly notable because legumes have traditionally been considered protein-rich foods rather than lipid-rich foods. The identification of GGA in both nuts and legumes indicates that its biosynthesis or accumulation in plants is not restricted to lipid-dense tissues. This finding suggests that GGA may have a broader metabolic or ecological role in plants.

In contrast, GGA was not detected in several tested items, including chickpeas, walnuts, hazelnuts, sesame seeds, dried mango powder, and parsley. Although negative findings are often underreported in nutritional science, they are nonetheless important. Such data provide critical reference points for delineating the dietary landscape of bioactive compounds like GGA. They also help define the practical boundaries of GGA availability in common foods. This, in turn, enables more accurate dietary intake assessments and supports the formulation of targeted dietary strategies.

The biological significance of dietary GGA intake lies in its emerging role as a regulator of hepatocyte stability. Recent studies have demonstrated that GGA can induce pyroptotic cell death via TLR4 signaling in hepatoma cells (4, 5).

Additionally, previous research has shown that hepatic GGA levels decline with age and are depleted during hepatocarcinogenesis. Importantly, oral GGA supplementation in mice significantly suppressed spontaneous liver tumor development (11), supporting its potential as a functional food component for liver cancer chemoprevention.

Together, these results provide novel insights into the food-based distribution of GGA and its potential contribution to human health. By identifying accessible and commonly consumed sources of GGA beyond turmeric, this study lays the groundwork for the development of diet-based preventive strategies targeting liver carcinogenesis and possibly other chronic conditions associated with lipid signaling or immune regulation. In addition to its anti-carcinogenic potential, GGA has been shown to exert reproductive benefits in mammals. A study in C3H/HeN mice reported that GGA supplementation during mating, pregnancy, and lactation significantly improved offspring survival and litter size (15). Moreover, a Japanese patent describes the application of GGA as a feed additive and a promoter for improving birth rates in mammals, suggesting broader physiological relevance beyond liver health (16). Supporting this notion, a recent comprehensive review summarized various dietary phytochemicals and their impacts on reproductive physiology and health (17). Collectively, these findings indicate that GGA’s physiological role may extend beyond tumor suppression to include developmental and reproductive functions, further highlighting the potential value of dietary GGA intake across the lifespan.

However, this study has several limitations. First, the sample size was relatively small (*n* = 3 per food item), which may not fully capture the variability within or between batches of food products available in the market. Second, the extraction method was optimized for lipophilic compounds and may have limited ability to detect conjugated or esterified forms of GGA. Third, the study evaluated only powdered commercial food products and did not assess the effects of food preparation or cooking, which may influence GGA content.

To address these gaps, future studies should explore a broader range of food matrices, processing conditions, and culturally diverse diets to better understand the dietary availability of GGA. Additionally, controlled dietary intervention studies in humans are needed to determine whether the consumption of GGA-rich foods leads to measurable increases in circulating or tissue GGA levels. Finally, epidemiological research linking dietary GGA intake with liver health outcomes would provide essential evidence for its relevance as a nutritional factor in cancer chemoprevention.

## Data Availability

The raw data supporting the conclusions of this article will be made available by the authors, without undue reservation.
