# Supplementary material for: Quantitative screening of geranylgeranoic acid in selected plant-based foods using LC/MS/MS
Source: Front Nutr. 2025 Aug 12;12:1652270. doi: 10.3389/fnut.2025.1652270 (PMC12378757; doi:10.3389/fnut.2025.1652270)
Supplement: Supplementary file 1 [file Data_Sheet_1.pdf]

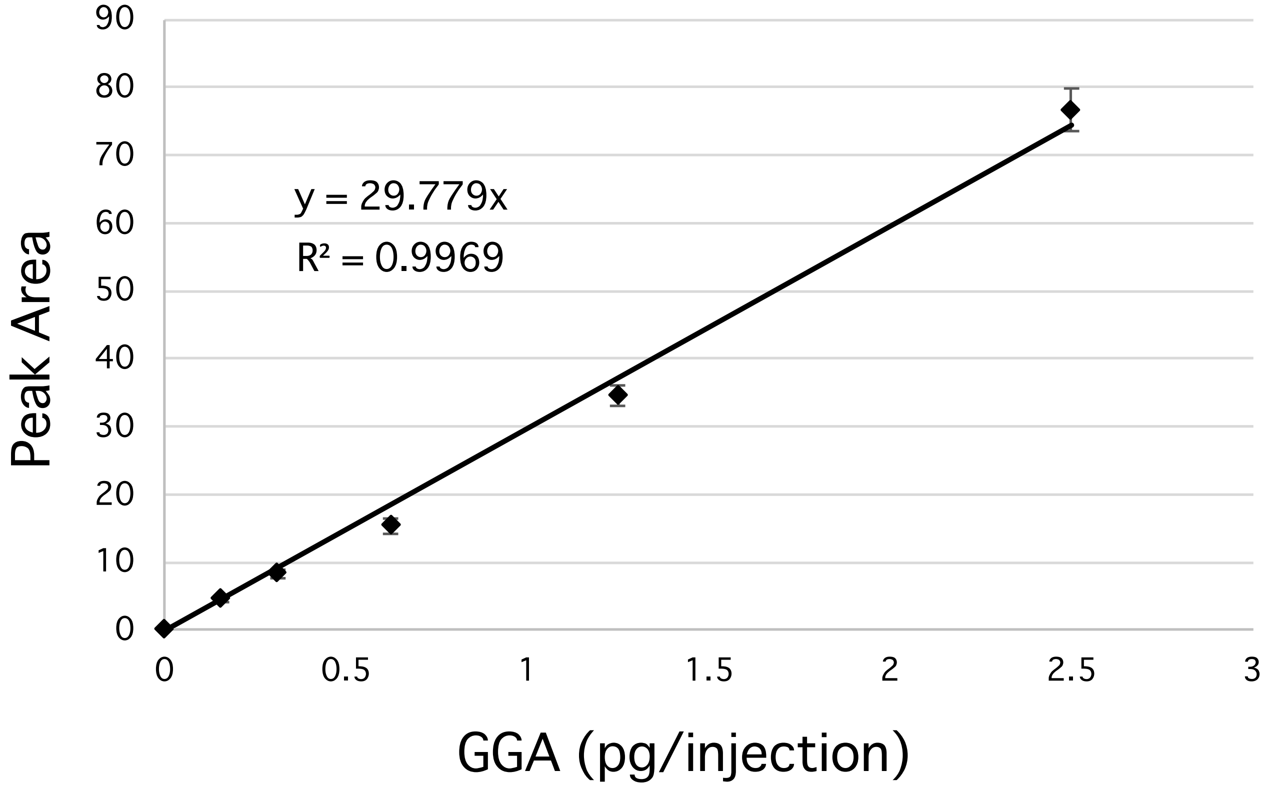

Figure S1. Calibration curve for the quantification of geranylgeranoic acid (GGA) using LC/MS/MS in MRM mode (transition: m/z 303 → 98). Standard solutions were prepared in the range of 0.15–2.5 pg/injection. The peak area (arbitrary units) was plotted against the injection amount, and the curve shows excellent linearity with a correlation coefficient (R^2) of 0.9969.

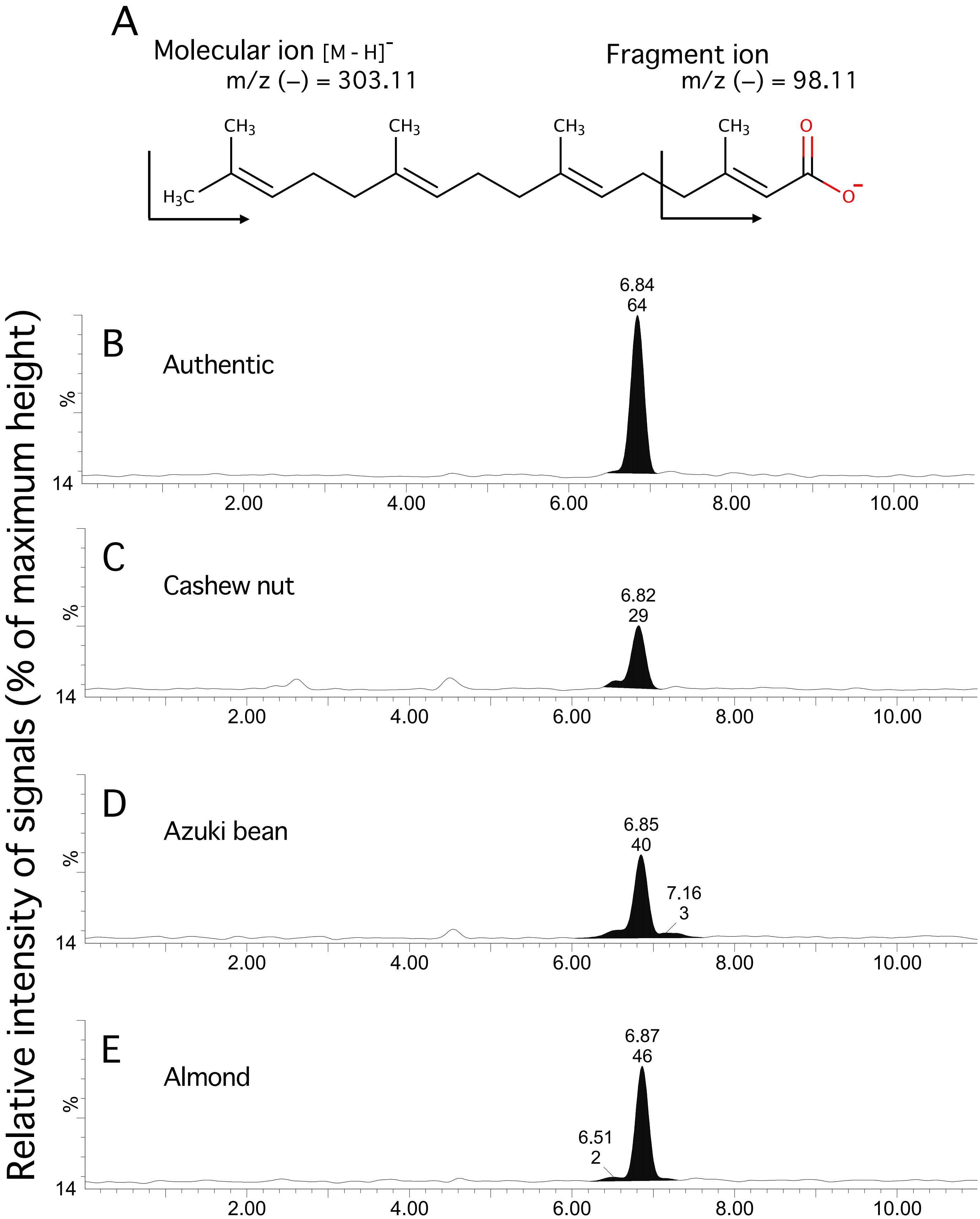

Figure S2.
A: Selected multiple reaction monitoring (MRM) transition used for the quantification of geranylgeranoic acid (GGA), showing the molecular ion (m/z 303.11) and its major fragment ion (m/z 98.11) in negative ion mode. The cleavage site is indicated in the chemical structure.
Representative MRM chromatograms of GGA in (B) cashew nut, (C)azuki bean and (D) almond samples. The MRM transition m/z 303.11 → 98.11 was monitored in negative ion mode. Chromatograms were obtained using a 10 μL injection volume. The retention time and peak shape are consistent with the authentic GGA standard.
